# Three-Dimensional Upper Body Kinematics and Inter-articular Kinematic Sequence During a Canoe Polo Throw

**DOI:** 10.3389/fspor.2021.777410

**Published:** 2021-12-15

**Authors:** Najoua Assila, Cyril Delavallade, Yoann Blache, Christian Berger-Vachon, Philippe Collotte, Sonia Duprey

**Affiliations:** ^1^Univ Lyon, Université Claude Bernard Lyon 1, Univ Gustave Eiffel, LBMC UMR_T9406, Lyon, France; ^2^Laboratoire de Simulation et de Modélisation du Mouvement, École de Kinésiologie et des Sciences de l'Activité Physique, Faculté de Médecine, Université de Montréal, Montreal, QC, Canada; ^3^Santy Paramedical Center, Lyon, France; ^4^Univ Lyon, Université Claude Bernard Lyon 1, Laboratoire Interuniversitaire de Biologie de la Motricité (EA 7424), Lyon, France; ^5^Centre Orthopédique Santy, Hôpital Privé Jean Mermoz, Lyon, France

**Keywords:** overarm motion, overhead sport, proximal-to-distal sequence, angular velocities, pelvis mobility

## Abstract

Canoe polo is an increasingly popular discipline requiring both kayaking and ball-handling skills. While the kinematics of the upper body during throw has been investigated for several overhead sports, the canoe polo throw has still to be studied. Therefore, the aim of this study is to analyze the canoe polo throw kinematics in terms of angles and inter-articular sequencing to understand its specificity. A secondary aim was to investigate whether adding pelvis mobility has an impact. Nineteen male players of canoe polo were equipped with reflective body markers for the throw analysis. They performed 5 throws with the pelvis fixed and 5 throws with additional pelvic mobility in rotation around a vertical axis. Inverse kinematics was performed with OpenSim providing pelvis, trunk, and glenohumeral rotations. Angular velocities were calculated to build the inter-articular sequences relative to these throws. Statistical parametric mapping was used to assess the effect of pelvis mobility on the throwing kinematics. Similar kinematics patterns as in other overhead sports were observed, however, a different inter-articular sequence was found for the canoe polo throw with a maximal angular velocity occurring sooner for the thorax in axial rotation than for the pelvis in rotation. While the limitation of rotation of the pelvis around a vertical axis has an influence on the pelvis and trunk kinematics, it did not modify the kinematic sequence.

## Introduction

Canoe polo is an increasingly popular kayaking discipline with World Championships held every 2 years since 1994. On a 35 × 20 m stretch of water, two teams of five players compete to score a goal (1.2 × 1 m) 2 m above the water. The playing pitch can be in open water or swimming pools. Players can grab and throw the ball by hand or using their paddle (https://www.canoeicf.com/disciplines/canoe-polo).

**Figure 1:**
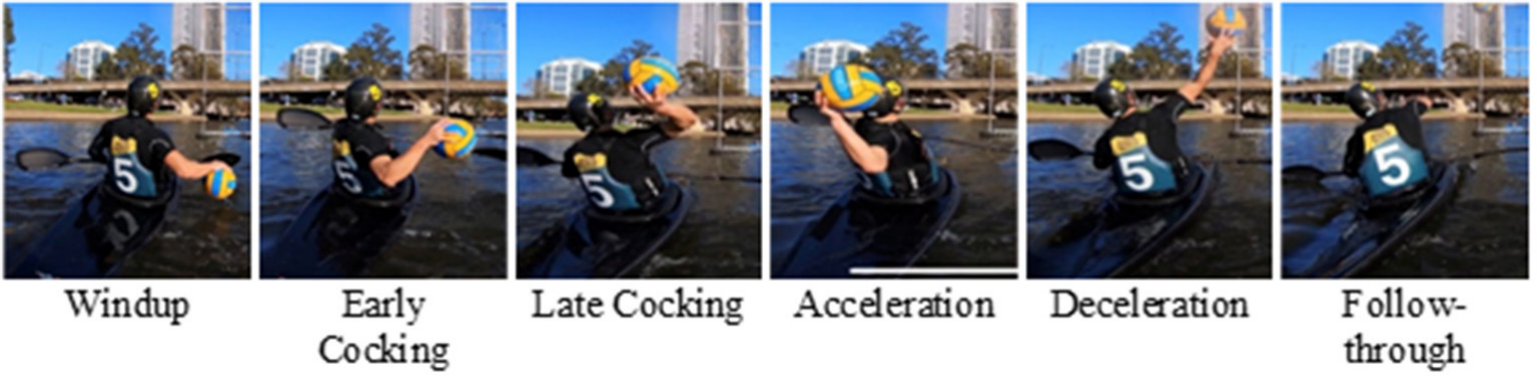
The throwing phases are illustrated during a canoe polo throw.

Canoe polo requires kayaking skills, particularly navigation and stabilization techniques, but also ball-handling skills to successfully manage throws toward the goal. In this sense, some comparisons can be established with other overhead sports, namely, water-polo, which shares the same ball and aquatic environment, handball, baseball, volleyball, or tennis because of the overhead motions. The kinematics of these latter have been widely documented (Anglin and Wyss, 2000; Park et al., 2002; Tillaar and van den Ettema, 2007; Martin et al., 2012; Kaczmarek et al., 2014; Gelber et al., 2018) and compared (Fleisig et al., 1996; Meriç et al., 2009; Wagner et al., 2014). In these studies, a thorough description of the overhead throwing motion in six phases has been provided: wind-up, early cocking, late cocking, acceleration phase, deceleration, and follow-through phase, which can also be observed in Canoe Polo (Figure 1). Furthermore, a similar inter-articular kinematic sequencing is generally observed in overarm motions (Fleisig et al., 1996; Marshall and Elliott, 2000; Fradet et al., 2004; van den Tillaar and Ettema, 2009; Wagner et al., 2014): a proximal-to-distal sequence with the maximum angular velocity being first reached by the pelvis rotation, then trunk rotation, trunk flexion, elbow extension and finally shoulder internal rotation and shoulder flexion, the angular velocity being the highest for the shoulder internal rotation. Despite these sequencing similitudes, discrepancies in maximal angles, angular velocities, and their timing were observed when comparing different overhead sports. For instance, averaged maximal shoulder internal rotations range from 4,520 ± 1,020°/s up to 5,580 ± 2,350°/s depending on the sport (Wagner et al., 2014). Furthermore, differences were observed between jumping vs. standing throws (Wagner et al., 2011), the contact with the floor enabling greater rotations of the trunk and pelvis and thus greater angular velocities for these segments.

In canoe polo, the athletes are in contact with the kayak at both footrest and seat level. Their legs are extended in the longitudinal direction of the boat throughout the throw, their pelvis mobility being constrained to avoid capsizing (Michael et al., 2009). These major constraints may influence the overhead motion. However, to our knowledge, their impact on canoe polo has not been studied yet. The impact of the mobility of the seat, however, has been reported to influence the paddling kinematics (Fohanno et al., 2011; Willmott and López-Plaza, 2014). The swivel seat that allows rotation about the vertical axis was designed to help the athlete's pelvic rotation and fluidify trunk rotation. In paddling, the use of a swivel seat reduced the rotation angles between the pelvis and the trunk and increased the kayaker's knee range of motion (Fohanno et al., 2011; Willmott and López-Plaza, 2014). As the swivel seat seems to modify the transfer of momentum from proximal to distal, we hypothesize that it might impact the kinematics of the canoe polo throw.

The aim of this study was to analyze the canoe polo throw kinematics in terms of angles [pelvis, trunk, and glenohumeral degrees-of-freedom (dof)] and inter-articular kinematic sequencing, considering the constraints imposed by the seated position. A secondary aim was to investigate whether adding pelvis mobility to the seated player (by enabling pelvis rotation) has an impact on its upper body kinematics and the sequencing. We hypothesize that the typical proximal-to-distal sequence found in overarm sports will be modified for canoe polo throws with the trunk reaching its maximal rotation velocity at the same time or before the pelvis. Our second hypothesis is that the pelvis axial rotation limitation is the major cause for this modified inter-articular sequence.

## Materials and Methods

### Data Collection

A group of 19 participants (29.4 ± 9.8 years old, 77.1 ± 10.6 kg, 1.78 ± 0.06 m) was recruited among elite male players of canoe polo (i.e., level of expertise greater or equal to the 4th French National League, Table 1). Only participants that did not suffer from any musculoskeletal pathology of the right or left shoulder during the past year were recruited. The protocol had obtained the agreement of the French ethical committee (CPP, protocol n° IDRCB: 2019-A00494-53), and each participant signed informed consent before the experiments. For all participants, the experiments were carried out during the winter break at the end of the season.

**Table 1:** The expertise of the recruited participants.

**Expertise**	**Number of participants**
1st French league	3
2nd French league	4
3rd French league	3
4th French league	9

Participants were equipped with 31 reflective body markers on their dominant arms (six markers on the scapula, four on the clavicle, nine on the humerus and forearm, and four on the hand), on their trunk (four markers), and pelvis (four markers; van Andel et al., 2009; Brochard et al., 2011; Jackson et al., 2012; Lempereur et al., 2014), Figure 2. Six reflective markers were used to track the ball.

**Figure 2:**
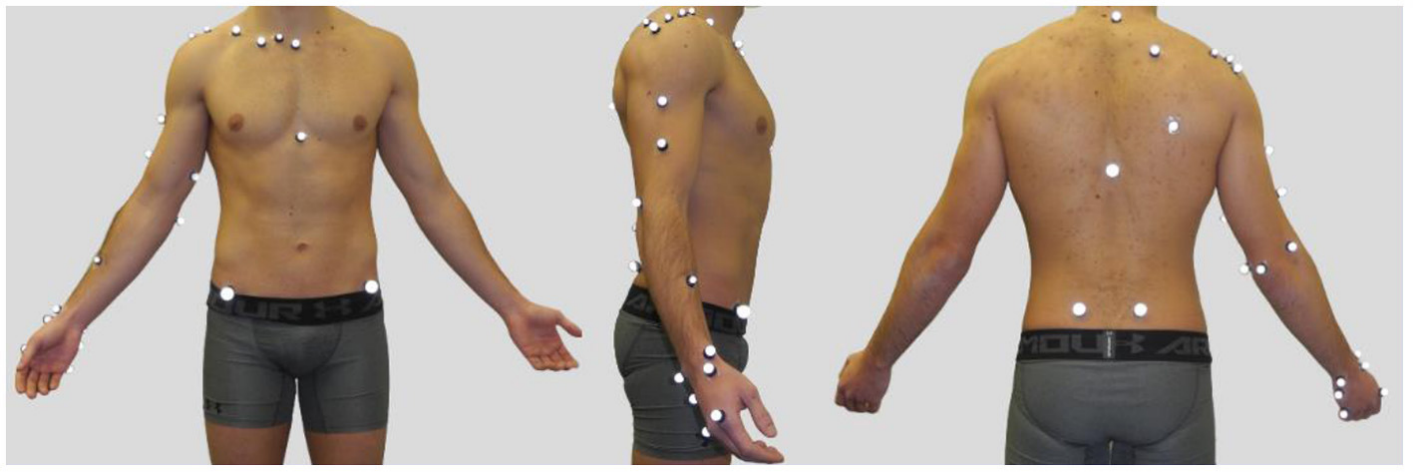
A participant equipped with reflective markers (left: anterior view, middle: lateral view, right: posterior view).

A Vicon MX-40 motion analysis system (Oxford Metrics Inc., Oxford, UK) was used to record the 3D trajectories of the markers. This system consists of 12 high-resolution cameras (4 mp) operating at a frequency of 200 Hz and positioned all around the player.

First, participants performed a 15 min-warm-up. It consisted in:

- 10 circumductions of the wrists (left and right at the same time), in two directions
- 10 flexion/extension motions of the elbows (left and right sides)
- 10 circumductions of the shoulder joint (left and right sides)
- 10 circumductions of the head, in two directions
- 10 external and internal rotations, the arm along the body, elbow 90°-flexed, with a rubber band (left and right sides).
- 10 external and internal rotations, shoulder 90°-abducted and elbow 90°-flexed, with a rubber band (left and right sides)
- 20 passes with a 3 kg weighted ball at 2 m distance
- maximal rotations of the trunk with 10-s hold while sitting on the seat, in 2 directions
- 10 throws while sitting on the seat.

This warm-up aimed at all the muscles of the shoulder joint complex and allowed the participants to increase their body temperature.

**Figure 3:**
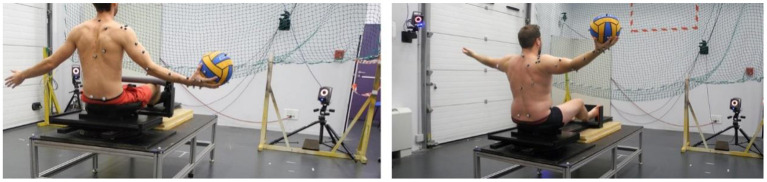
Illustrations of the 2 throwing configurations. Left: the pelvis is held immobile since the pelvis support cannot rotate and knees motions are prevented; right: the pelvis support can freely rotate, and the knee supports have been removed.

After this warm-up, participants were asked to take a static pose in the anatomical posture (Figure 2). Then, they were asked to sit in a mock-up boat to perform 10 throwing motions as powerful as possible, within the goal. They performed the following:

- 5 throws with the pelvis held immobile in the frontal plane. The pelvis posture was maintained by both immobilizing the seat axis of rotation and the use of knee supports (Figure 3, left).
- 5 throws with a free-to-rotate pelvis, initially positioned in the front plane (Figure 3, right).

A 1-min rest between throws was given to the player to recover, extra rest time was given upon request.

### Data Processing

**Figure 4:**
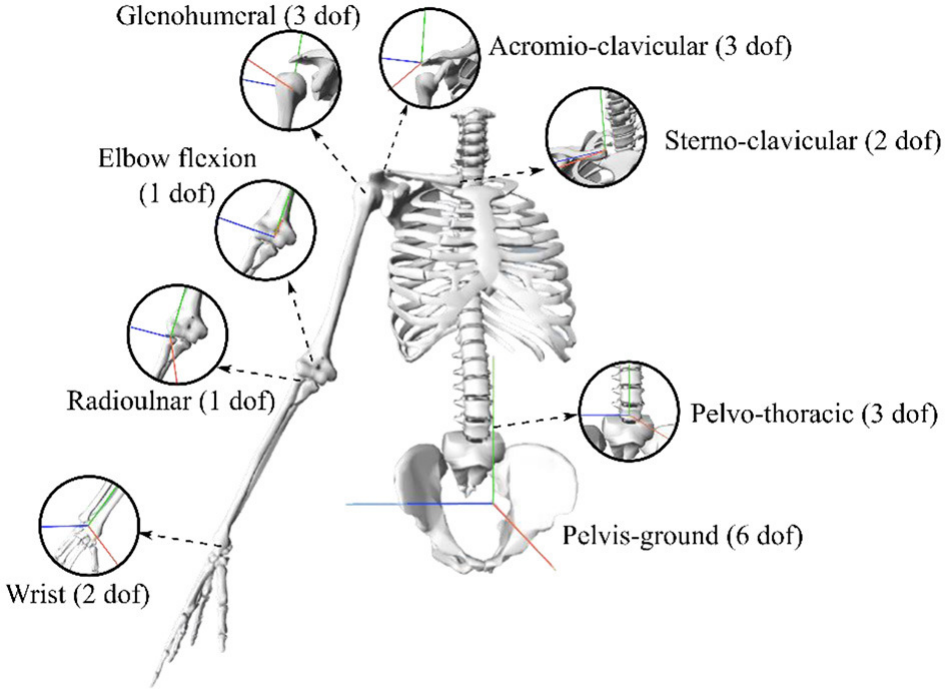
Kinematic model of the upper body.

Skeletal computations were carried using OpenSim (Stanford University, USA; Delp et al., 2007; Seth et al., 2018). A generic model derived from Wu et al. (2016) with the addition of the pelvis was used. The model was first scaled anthropometrically according to the positions of the markers on the bony landmarks in the anatomical pose. In this pose, the virtual markers were then placed to replicate the positions of the experimental markers. The choice of the kinematic model is key since the kinematic constraints can influence the results of the optimization (Naaim et al., 2017). Here, it was decided to choose the kinematics constraints that were the most used among the many existing modeling options (Figure 4) (Duprey et al., 2017): 6 dof at the pelvis (i.e., the pelvis is a free body), 3 dof at the pelvo-thoracic joint (i.e., a ball and socket joint), 2 dof at the sternoclavicular joint (i.e., 2 hinge joints), 3 dof at the acromioclavicular joint (i.e., a ball and socket joint), 3 dof at the glenohumeral joint (i.e., a ball and socket joint), 2 dof at the elbow (i.e., 2 hinge joints), and 2 dof at the wrist (i.e., 2 hinge joints).

The generalized coordinate values were predicted during the dynamic trials using the inverse kinematics tool in Opensim, namely, at each time frame the algorithm seeks to find the coordinate values set that minimizes the sum of weighted squared errors between the experimental and model markers, https://simtk.org/frs/?group_id=91.

In the rest of the study, we considered the following dof and their velocities:

- 3D angles of pelvis vs. the fixed reference coordinates system: pelvis rotation (rotation around a vertical axis), pelvis obliquity (lateral rotation), pelvis tilt (anteroposterior rotation).
- the 3D angles of the thoraco-pelvis joint: trunk flexion-extension (anteroposterior rotation), trunk lateral flexion, trunk axial rotation (trunk rotation around a vertical axis).
- the 3D angles of the glenohumeral joint: glenohumeral plane of elevation, glenohumeral elevation, and glenohumeral axial rotation.

A schematic of these dofs can be found in Supplementary Figure 1. Furthermore, the ball velocity was also calculated and presented from the onset of the throw-up to the ball release. The ball release moment was defined when the distance between the hand markers centroid and that of the ball was 10 mm higher than its mean during the first phase of the throw.

To ease the comparisons, the throwing trials were all synchronized by setting the ball release at 80% of the trial. The beginning of the trial was defined as the onset of the trunk rotation around the vertical axis. The end of the trial matched with the trunk returning to its initial rest position. For some trials, the participants did not return directly to their initial trunk orientation, thus the end was defined when the glenohumeral axial rotation returned to its initial value. The time-series outcome measures are reported in the normalized trial space (and not in time). The duration of the 3 main phases - arm cocking (i.e., grouping wind-up, early and late cocking), acceleration phase, and post ball release (i.e., grouping deceleration and follow-through phases) were calculated as in previous studies (Wagner et al., 2010, 2011). The arm coking phase is the time lag between the beginning of the motion (i.e., the onset of the trunk rotation) and the moment when the angular acceleration of the trunk rotation is maximal (Wagner et al., 2011). The arm acceleration is the time lag between the end of the arm cocking phase and ball release. The post-ball release starts from the ball release and goes up to the end of the motion.

The inter-articular sequence was defined by identifying the moment the angular velocities are at their maxima and was presented with respect to the moment the glenohumeral axial rotation velocity was at its maximum (which corresponds to zero). The elbow flexion was also included in the sequence, as it plays an important role in the throw motion, mainly to reduce the moment arm for the glenohumeral axial rotation and is usually reported in existing proximal-to-distal sequence (Fleisig et al., 1996; Marshall and Elliott, 2000; van den Tillaar and Ettema, 2009; Wagner et al., 2014).

### Statistics

The statistical analysis aimed at comparing the dof values and their velocity histories, as well as the ball velocities, during throws with the fixed pelvis vs. free pelvis. These two conditions were compared using statistical non-parametric mapping implemented in the spm1d Python library (Pataky et al., 2015) (https://spm1d.org/References.html). A non-parametric paired *t*-test was used to evaluate the effect of the pelvis mobility, as non-parametric testing is more robust to non-normal data (Pataky et al., 2015). Statistical significance was fixed at 0.05. The family-wise error rate was controlled using a Bonferonni correction across the 9 dof of interest.

Furthermore, we tested the effect of pelvic mobility on the inter-articular sequence using a non-parametric implementation of an ANOVA (repeated measures on pelvis mobility; Nichols and Holmes, 2002) with the moments of maximum velocity (for pelvic rotation, trunk axial rotation, and flexion, the glenohumeral plane of elevation, elevation, and axial rotation, and elbow flexion) and pelvis mobility (free vs. fixed) as factors. In case of significance, *post-hoc t*-tests were applied using a Bonferonni correction across the number of tests.

## Results

**Figure 5:**
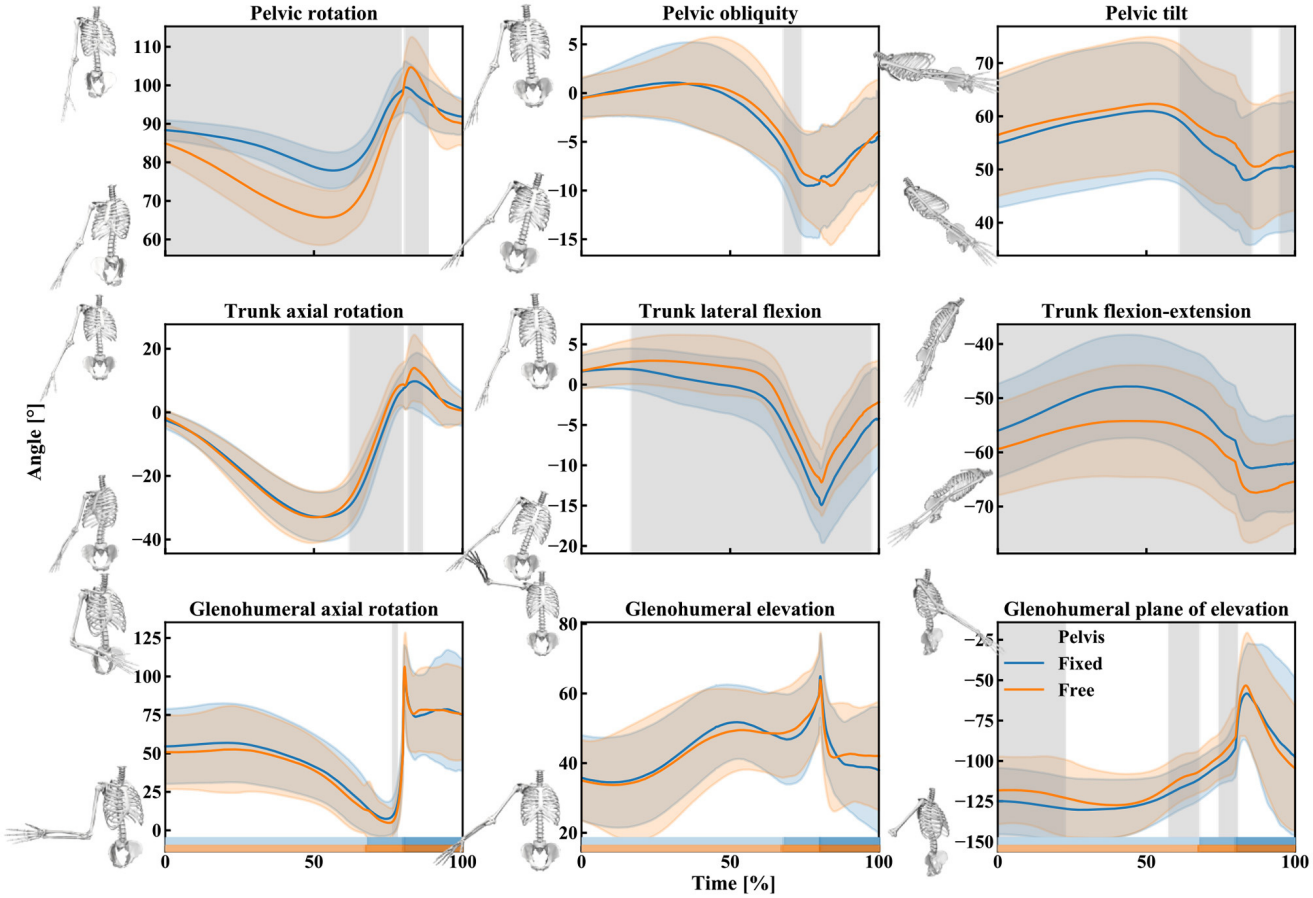
Mean (lines) and standard deviation (slightly transparent corridors) of the pelvis, trunk, and shoulder rotation angles during the free pelvis (orange) and fixed pelvis (blue) throw. A gray area indicates the presence of a significant effect of the pelvis condition (free vs. fixed) over time. The rectangles above the x-axis indicate the different phases of the throw for the two pelvis conditions (from brightest to darkest: cocking phase, acceleration phase, and post ball release; orange: free pelvis, blue: fixed pelvis).

**Figure 6:**
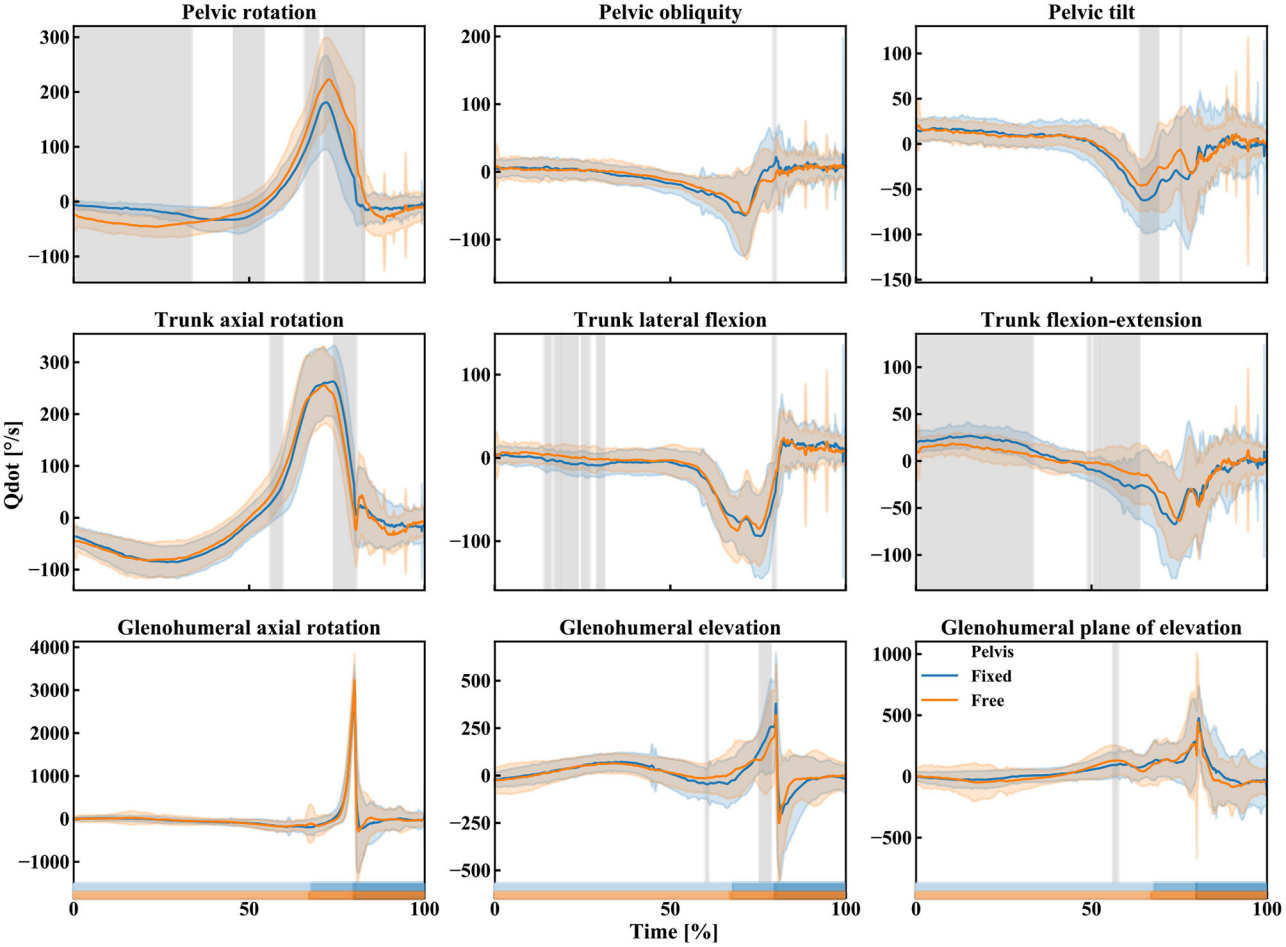
Mean (lines) and standard deviation (slightly transparent corridors) of the pelvis, trunk, and shoulder angular velocities during the free pelvis (orange) and fixed pelvis (blue) throw. A gray area indicates the presence of a significant effect of the pelvis condition (free vs. fixed) over time. The rectangles above the x-axis indicate the different phases of the throw for the two pelvis conditions (from brightest to darkest: cocking phase, acceleration phase, and post ball release; orange: free pelvis, blue: fixed pelvis).

The canoe-polo throw starts with the pelvis and trunk rotating backwards [the cocking phase, from 0 to 68.01 ± 2.93% (fixed pelvis) vs. 67.22 ± 3.37% (free pelvis)], the shoulder is externally rotated (Figure 5). Then, during the acceleration phase, shoulder internal rotation (glenohumeral axial rotation) occurs resulting in shoulder angular velocities up to about 3,500°/s (Figures 5, 6).

**Figure 7:**
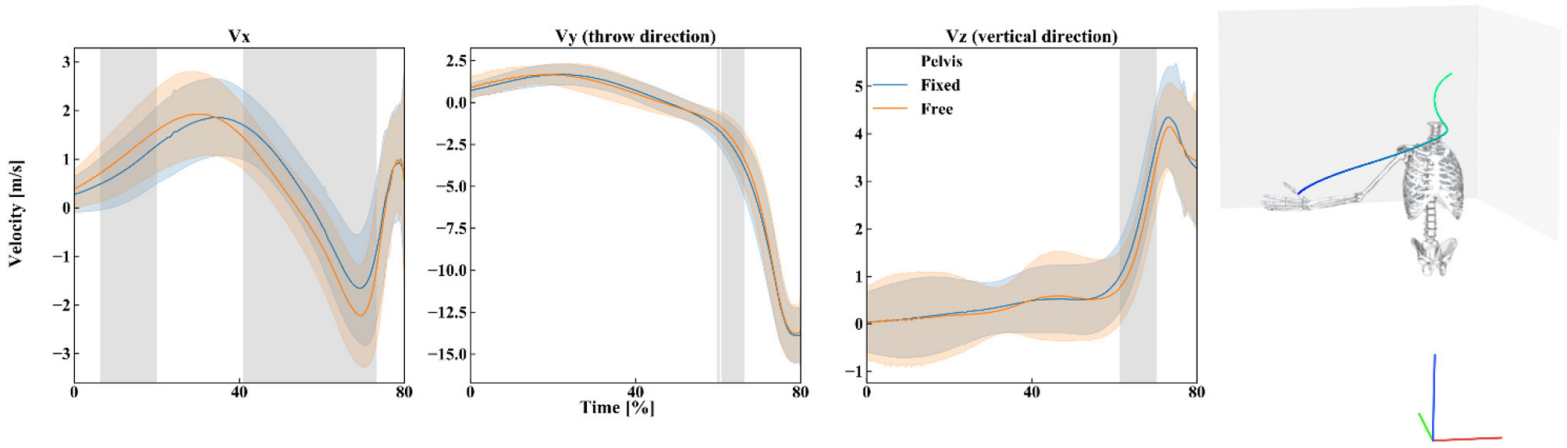
Left: Mean (lines) and standard deviation (slightly transparent corridors) of the ball linear velocity in the three directions of space during the free pelvis (orange) and fixed pelvis (blue) throw. A gray area indicates the presence of a significant effect of the pelvis condition (free vs. fixed) over time. Right: Schematic of the position of the participant in the reference frame of the ball velocity (red: x-axis, green: y-axis, blue: z-axis), and the mean ball trajectory. The time scale is represented with the trajectory color (blue for 0% and green at ball release).

**Figure 8:**
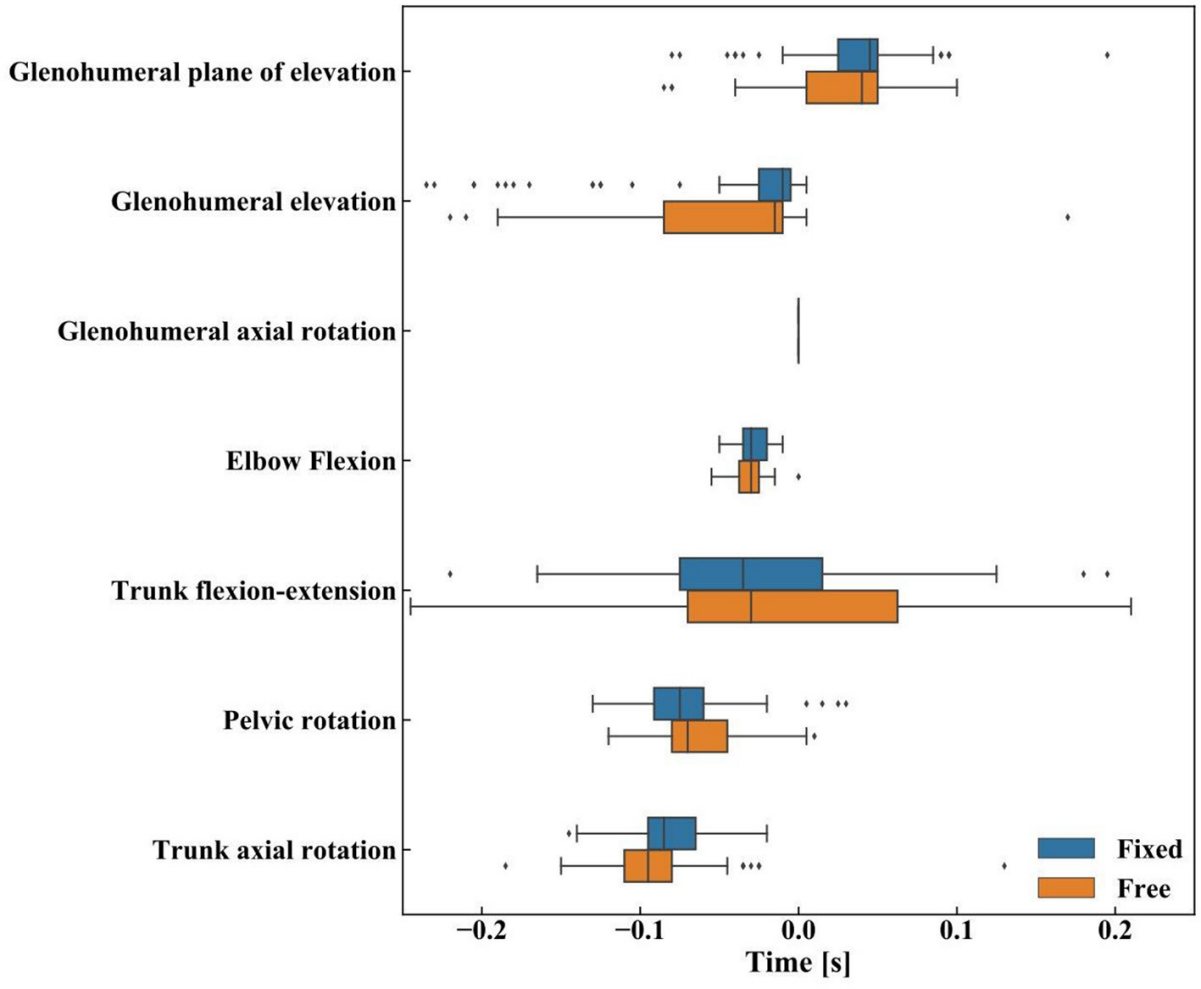
The inter-articular sequence during canoe-polo throws: boxplot of the maximal angular velocities for the upper body degrees of freedom with median (vertical lines), first-third interquartile (IQR) range (bars), minimum-maximum range [*Q*1 − 1.5*IQR, Q3 + 1.5*IQR] (whiskers). Data beyond the whiskers are considered outliers (points).

Qualitatively, the trunk axial rotation seems to reach its maximal angular velocity first, followed closely by the pelvis rotation, then the trunk flexion-extension, elbow flexion, and the glenohumeral dofs (Figures 6–8).

The kinematics obtained during the two different types of throws (fixed or free pelvis) differ for the pelvis and trunk rotations (pelvis rotation, trunk flexion-extension, and lateral flexion mainly) but only slightly for the shoulder (Figure 5, the details of the *t*-statistic and *p*-values can be found in Supplementary Figure 2). The significant differences observed for the pelvis rotation confirm that the participants actually utilized the axial rotation allowed by the seat: when free, the pelvis rotates more backward (about 20° on average) during the cocking phase (Figure 5). As for the trunk, in the “free pelvis” configuration, its flexion was larger (about 10° on average) as well as its lateral flexion (a few degrees on average). However, despite these kinematics differences, the players reached statistically similar magnitudes of angular velocities at the shoulder (~3,500°/s of glenohumeral axial rotation, Figure 6; the details of the *t*-statistic and *p*-values can be found in Supplementary Figure 3). Furthermore, no significant differences were found for the ball release speed (Figure 7, the details of the t-statistic and p-values can be found in Supplementary Figure 4), despite some differences being observed at the previous phases of the throw.

The two-way ANOVA yielded a significant effect for the timing of the maximal angular velocities (F = 30.035, *p* < 0.001) but didn't show any significant effect of the pelvis mobility (*F* = 1.056, *p* = 0.365), nor of its interaction with the timing (*F* = 2.098, *p* = 0.087, Figure 8). The *post-hoc* analysis confirmed significant differences between the timing of the maximal angular velocities for all dof combinations (*p* < 0.001), except for the pairs: trunk flexion-extension vs. elbow flexion, trunk flexion-extension vs. glenohumeral axial rotation, trunk flexion-extension vs. glenohumeral elevation, and elbow flexion vs. glenohumeral elevation (the detail of the *p*-values can be found in Supplementary Figure 5).

## Discussion

This study investigated the canoe-polo throw and the effects of the pelvis rotation mobility on the kinematics of the upper body and the inter-articular coordination. As hypothesized, the typical proximal-to-distal sequence found in overhead sports is modified for canoe polo throws. However, the pelvis axial rotation limitation isn't the major cause for this modification, as we initially thought.

First, similitudes can be observed between canoe polo throws and other overhead sports. The same phases (wind-up, early cocking, late cocking, acceleration phase, deceleration, and follow-through phase) are found with qualitatively similar kinematics: the pelvis and trunk rotate backwards during the cocking phase, then during the acceleration phase the shoulder internally rotates (Wagner et al., 2014). The maximal shoulder velocity obtained here was about 3,500°/s in accordance with the magnitudes listed in the literature (from 4,520 ± 1,020°/s up to 5,580 ± 2,350°/s). However, the proximal-to-distal sequence is modified during a canoe polo throw with respect to other sports, confirming our first hypothesis. Here, the pelvis kinematics being limited, it reaches its maximal velocity in rotation slightly later than the trunk in rotation: on average about 0.018's later, while Wagner et al. (2014) reported that the maximal angular velocity occurs sooner for the pelvis rotation vs. the trunk rotation: 0.029 s sooner for team handball throw, 0.036's for a tennis serve and 0.05's for the volleyball spike. The rest of the sequence was yet the same as reported in the literature: trunk flexion, elbow extension, and finally shoulder dofs (Fleisig et al., 1996; Marshall and Elliott, 2000; van den Tillaar and Ettema, 2009; Wagner et al., 2014).

We also hypothesized that the major reason for these sequencing discrepancies would be the mobility of the pelvis in rotation. When comparing the upper body kinematics for two pelvis mobility configurations (free or fixed pelvis) using spm, significant differences were actually observed for the pelvis rotation, trunk flexion-extension, and trunk lateral flexion mainly. It might seem trivial to observe pelvis rotation discrepancies. However, this result is actually useful to confirm that canoe polo players really capitalize on the additional pelvis rotation around the vertical axis. With this mobility addition, the pelvis could rotate more backwards, and the trunk dofs were larger (flexion and lateral flexion). These significant differences visible for the trunk but not for the shoulder show that it is the thoraco-pelvis joint—and the trunk itself—that adapt to the pelvis mobility rather than the shoulder. This is in accordance with the results obtained when using the swivel seat for paddling: while its use influenced the rotation angles between the pelvis and the trunk, the upper limb kinematics were less affected by the choice of the seat (Fohanno et al., 2011; Willmott and López-Plaza, 2014). It is also in accordance with the analysis from Murta et al. (2020), who reported the impact of a pelvic mobility reduction on the trunk kinematics but not on the shoulder kinematics. The significant differences observed for the glenohumeral plane of elevation dof were limited to a couple of degrees (<10°) and for limited time periods (compared to the pelvis and trunk significant differences). They may be explained by trunk geometry modification (lumbar and thoracic lordosis or kyphosis) generating scapula posture modifications and thus glenohumeral kinematics changes (Suzuki et al., 2019). However, this assumption should be taken with caution since the trunk geometry wasn't specifically measured here.

Thus, adding pelvis mobility clearly had an impact on the upper body kinematics, however, the inter-articular sequence was not modified. The sequence “trunk axial rotation, pelvis rotation, trunk flexion-extension, and then upper limb dofs” was not changed, which might indicate that the limitation in pelvis axial rotation was not responsible for this specific sequence. However, even with the added pelvis mobility, the maximum pelvic rotation (−20°) in reference to the position at rest remains smaller than those observed for tennis (−99°), handball (−43°), or volleyball (−48°) (Wagner et al., 2014). Thus, it is possible, that despite the pelvis enabled mobility, the legs position still imposes a significant constraint on the pelvic rotation. In other sports with an overarm throw, the proximal-to-distal sequence ensures an optimal transfer of energy from the lower body to the ball. The position of the legs, as well, as the double contact with the kayak (seat and footrest) might disturb the energy chain resulting in a different technique to increase the throw velocity. Further studies that measure the forces at the footrest and the seat rotation axis will be needed to understand the reason for the shift in this sequence.

The pelvis mobility impacted the ball velocity. Particularly, the increased linear velocity in the lateral direction might be related to the increased pelvis rotation and trunk flexion. However, as the ball reached the release point, no statistically significant difference was observed. This indicates that pelvis mobility might not impact the trajectory, nor the distance traveled by the ball after the throw. However, the absence of a difference might be the result of the players' expertise. Indeed, as the players are trained to control their throws despite the boat instability, they might have used a similar technic to counter the effect of the swivel seat. It would be interesting for future studies, to evaluate the effect of the pelvis mobility on the ball velocity when thrown with the paddle, as the increased moment arm might further exacerbate the effect of the increased trunk flexion.

Indeed, our protocol had some limitations. In particular, the mobility of the boat on the water was not reproduced, although it could generate larger core muscle actions from the canoe polo player and thus change the thoraco-pelvis mobility. To provide a thorough investigation, it would be interesting to study the impact of boat instability by performing field studies. Another limitation is that the starting posture before the throw isn't the initial natural position of the kayak polo throw since players were asked to take an anatomical posture so the markers could easily be identified. The right position would be hand in pronation and ball in the water. Furthermore, the ball and hand were not wet which may have affected the throw for some participants. Third, although it wouldn't change the inter-articular sequencing presented here, including the scapula-thoracic joint could have permitted to see if kinematics differences were generated at this joint when the pelvis was free or fixed. Additionally, the order of conditions was not randomized across participants, which might have introduced some bias. However, the variability across participants for each condition was comparable which indicates that the bias was negligible. Finally, it would have been interesting to test a larger panel of players from different levels and to include female players to broaden the present conclusions.

As for the modeling limitations, the trunk model could be improved since it is here modeled by a single segment (Wu et al., 2016). The prospects would be to perform the kinematic analysis with a more precise model of the trunk since the lack of rotation of the spine at different stages might affect the shoulder kinematics.

In terms of short-term perspectives, implementing a dynamic analysis (i.e., inverse dynamics and static optimization to estimate muscular forces) might be interesting. Analyzing articular torques and muscular forces for free and fixed pelvis may shed more light on differences at the shoulder. Indeed, the absence of major significant glenohumeral differences does not necessarily mean an absence of difference in muscles recruitment. Also, a power analysis based on the kinetic chain of the different segments involved in the throw, determined by the amplitude of the articular torque and the angular velocity as described by Martin et al. (2014), analyzing the tennis service, may be of interest.

## Conclusion

This study provides a better understanding of the kinematics of the upper body during a canoe polo throw. While similar general patterns as in other overhead sport throws are found, a different kinematic sequence is obtained for the canoe polo throw with a maximal angular velocity occurring sooner for the thorax axial rotation than for the pelvis rotation.

Investigations on the pelvis mobility of the seated player showed that the limitation of rotation of the pelvis around a vertical axis had an influence mainly on the pelvis and thorax kinematics but not on the shoulder ones and that it was not the cause for this modified kinematic sequence. Further studies will be needed to shed a light on the reason for this specific kinematic sequencing.

## Data Availability Statement

The raw data supporting the conclusions of this article will be made available by the authors, without undue reservation.

## Ethics Statement

The studies involving human participants were reviewed and approved by n° IDRCB: 2019-A00494-53 from French CPP. The patients/participants provided their written informed consent to participate in this study.

## Author Contributions

PC and CD: conceptualization. SD, CB-V, and YB: methodology. NA and YB: software. CD, CB-V, and SD: investigation/experimentations. CD and NA: writing—original draft preparation. SD, YB, CB-V, and PC: writing—review and editing. SD and YB: supervision. PC: funding acquisitions. All authors contributed to the article and approved the submitted version.

## Funding

This study was funded by Ramsay Santé, France.

## Conflict of Interest

The authors declare that the research was conducted in the absence of any commercial or financial relationships that could be construed as a potential conflict of interest.

## Publisher's Note

All claims expressed in this article are solely those of the authors and do not necessarily represent those of their affiliated organizations, or those of the publisher, the editors and the reviewers. Any product that may be evaluated in this article, or claim that may be made by its manufacturer, is not guaranteed or endorsed by the publisher.
