# Supplementary material for: Three-Dimensional Upper Body Kinematics and Inter-articular Kinematic Sequence During a Canoe Polo Throw
Source: Front Sports Act Living. 2021 Dec 15;3:777410. doi: 10.3389/fspor.2021.777410 (PMC8714653; doi:10.3389/fspor.2021.777410)
Supplement: Supplementary file 1 [file Data_Sheet_1.docx]

Supplementary Material

# Supplementary Figures

**
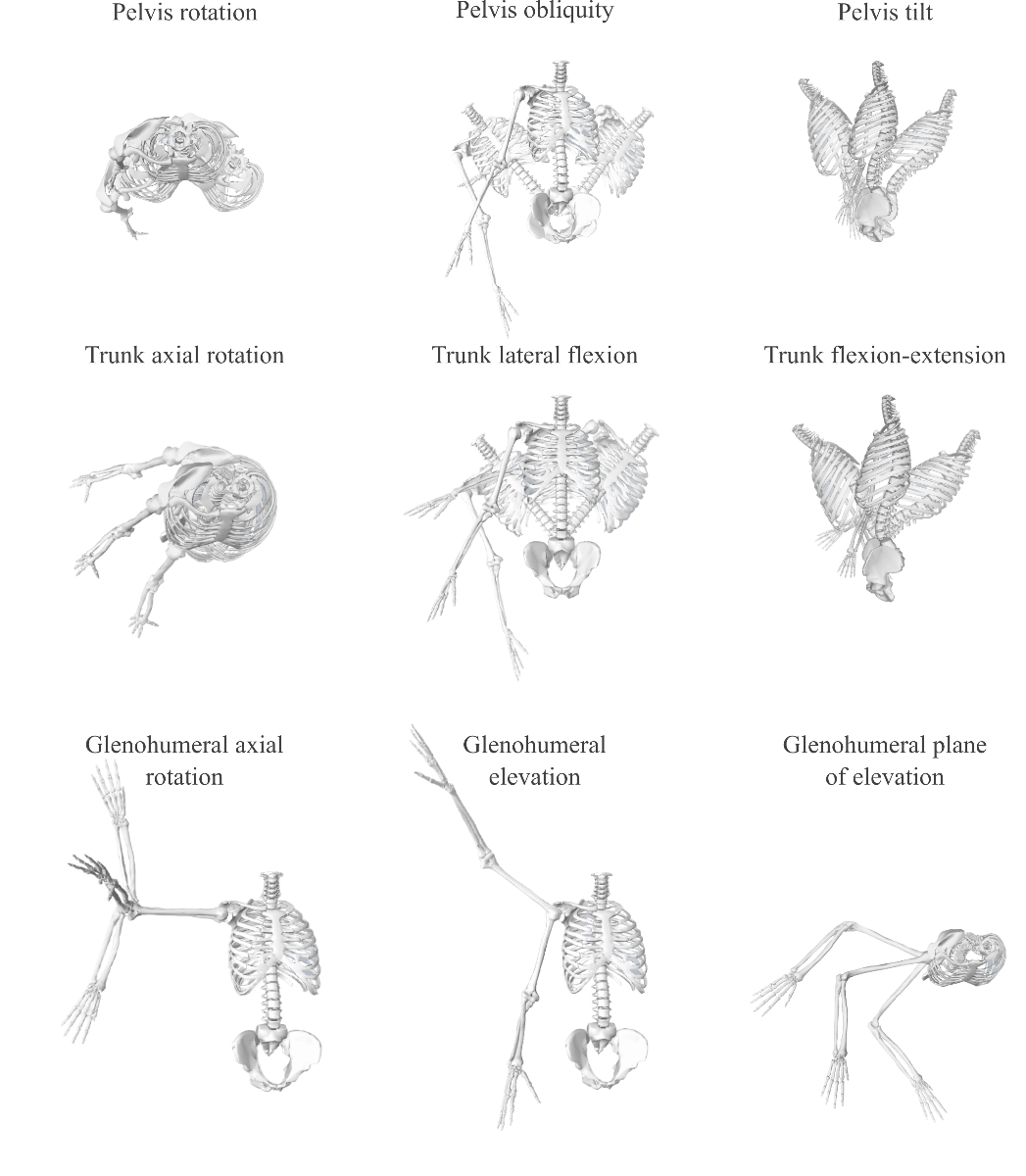
**

**Supplementary Figure 1.** Schematic of the degrees of freedom that define the orientation of the pelvis versus the fixed reference as well as the kinematics of the thoraco-pelvis and glenohumeral joints.

**
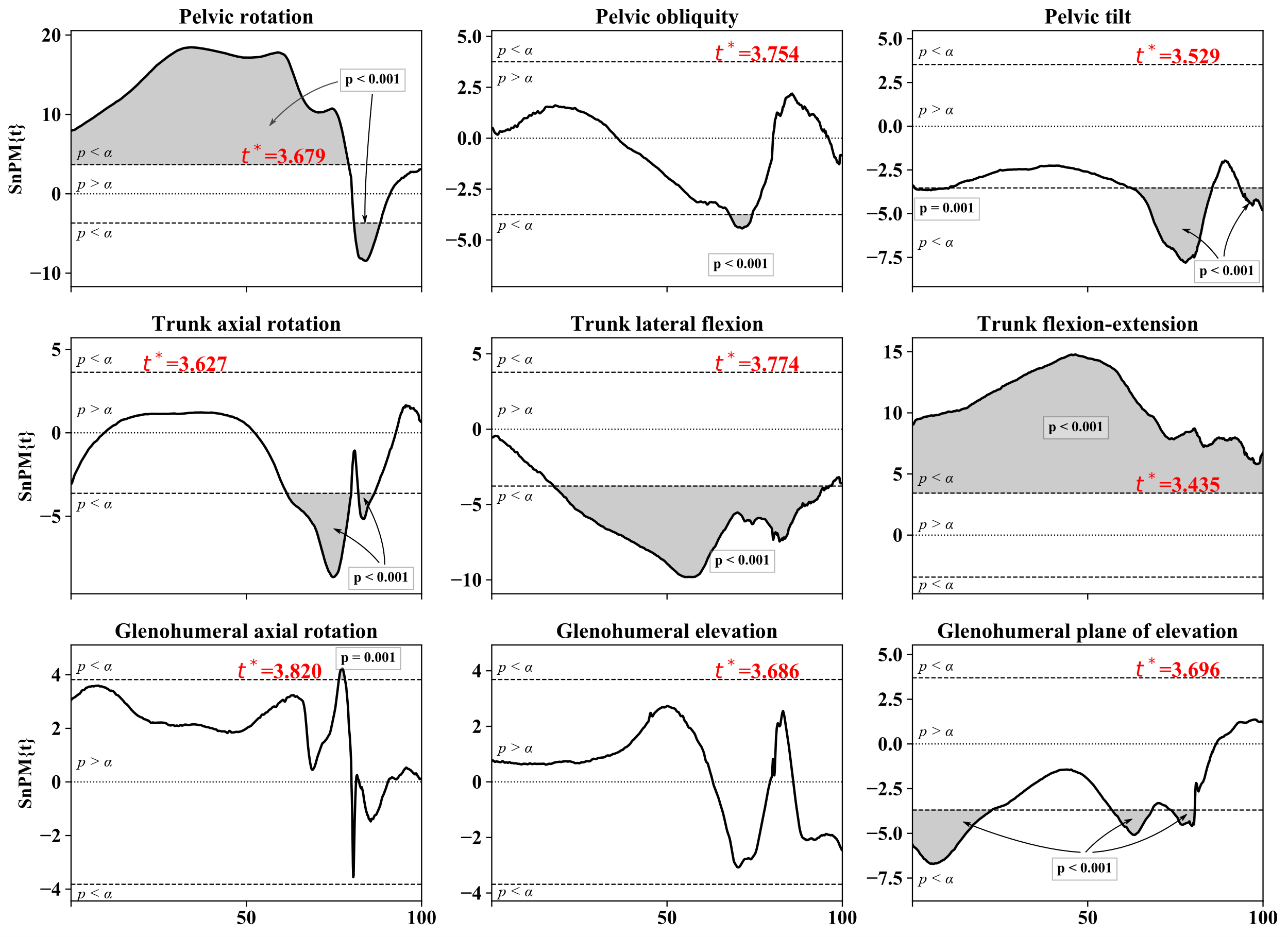
**

**Supplementary Figure 2.** Statistical non-parametric mapping t values from the t-test evaluating the effect of pelvis mobility on dof angles. Dotted horizontal lines represent the critical threshold relative to the significance level. Shaded areas represent the presence of a statically significant difference.

**
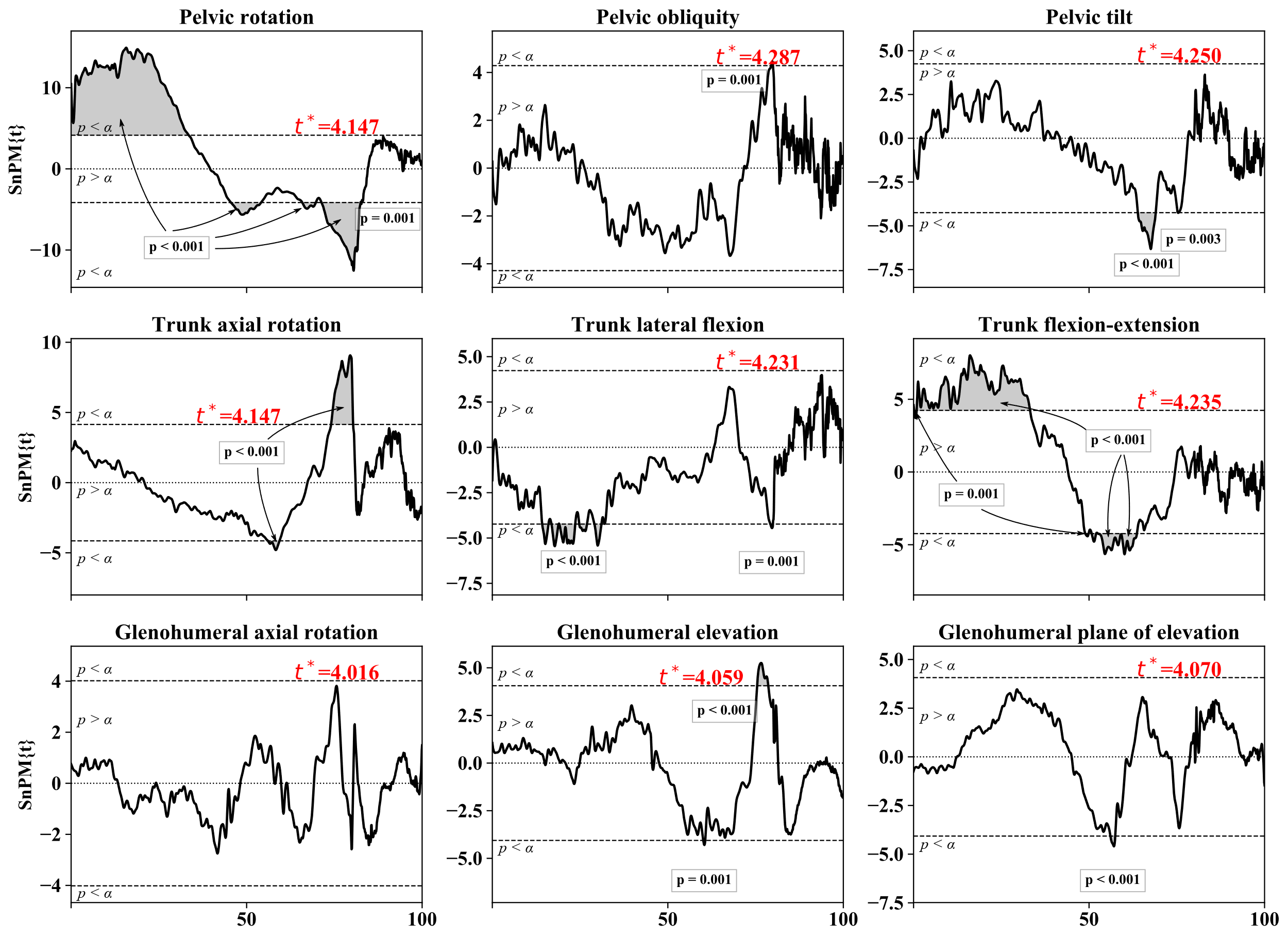
**

**Supplementary Figure 3.** Statistical non-parametric mapping t values from the t-test evaluating the effect of pelvis mobility on dof angular velocities. Dotted horizontal lines represent the critical threshold relative to the significance level. Shaded areas represent the presence of a statically significant difference.


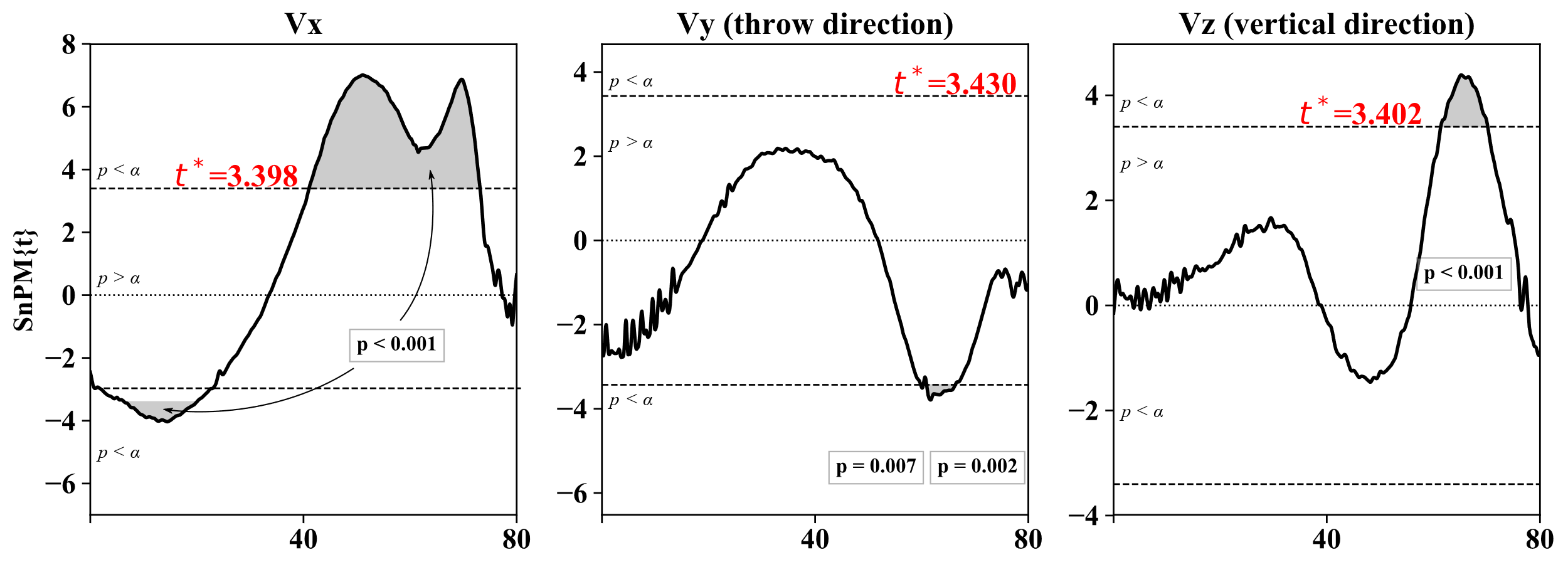


**Supplementary Figure 4.** Statistical non-parametric mapping t values from the t-test evaluating the effect of pelvis mobility on the ball linear velocities in time. Dotted horizontal lines represent the critical threshold relative to the significance level. Shaded areas represent the presence of a statically significant difference.


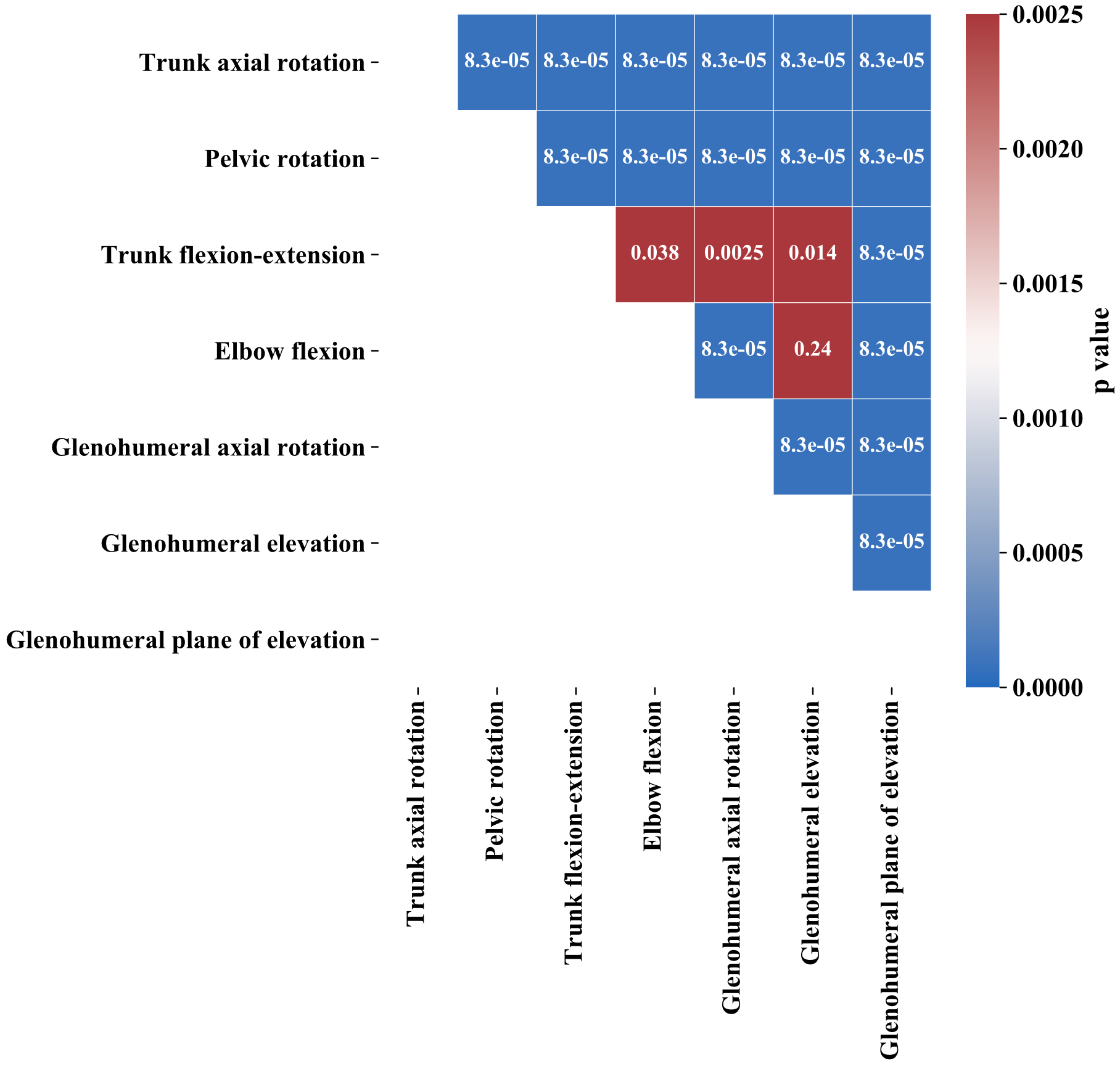


**Supplementary Figure 5.** P-values of the post-hoc analysis. Red values showcase non-significant differences between the time of maximal angular velocity of the row dof and the column dof.
